# Phylogenetic Analysis of European Brown Hare Syndrome Virus Strains from Poland (1992–2004)

**DOI:** 10.3390/v13101999

**Published:** 2021-10-05

**Authors:** Andrzej Fitzner, Ewa Kwit, Wiesław Niedbalski, Ewelina Bigoraj, Andrzej Kęsy, Artur Rzeżutka

**Affiliations:** 1 Department of Foot and Mouth Disease, National Veterinary Research Institute, ul. Wodna 7, 98-220 Zduńska Wola, Poland; wieslaw.niedbalski@piwet.pulawy.pl (W.N.); andrzej.kesy@piwet.pulawy.pl (A.K.); 2 Department of Food and Environmental Virology, National Veterinary Research Institute, Al. Partyzantów 57, 24-100 Puławy, Poland; ewa.kwit@piwet.pulawy.pl (E.K.); ewelina.bigoraj@piwet.pulawy.pl (E.B.)

**Keywords:** EBHSV, European hare, lagoviruses, phylogenetic relationships, evolution

## Abstract

European brown hare syndrome (EBHS) is lethal to several species of free-living hares worldwide. The genetic characterization of its virus (EBHSV) strains in European circulation and epidemiological knowledge of EBHSV infections is not yet complete. The study determined the nucleotide sequences of the genomes of EBHSV strains from Poland and analyzed their genetic and phylogenetic relationships to a group of hare lagoviruses. The genome of five virus strains detected in Poland between 1992 and 2004 was obtained by RT-PCR and sequencing of the obtained amplicons. The genetic relationships of the EBHSV strains were analyzed using the full genome and VP60 gene sequences. Additionally, the amino acid sequence of the VP60 gene was analyzed to identify mutations specific to recognized EBHSV subgroups. Partial amplification of the virus open reading frame (ORF)1 and ORF2 regions obtained nearly complete nucleotide genome sequences of the EBHSV strains. Phylogenetic analysis placed them in a GII.1 cluster with other European strains related to nonpathogenic hare caliciviruses. VP60 gene analysis allocated these EBHSV strains to the G1.2, G2.2–2.3 or G3 virus genetic groups. The amino acid sequence differences in the entire genome ranged from 1.1 to 2.6%. Compared to a reference French EBHSV-GD strain, 22 variable amino acid sites were identified in the VP60 region of the Polish strains, but only six were in VP10. Single amino acid changes appeared in different sequence positions among Polish and other European virus strains from different genetic groups, as well as in VP10 sequences of nonpathogenic hare caliciviruses. The results of the study showed a high genetic homogeneity of EBHSV strains from Poland despite their different location occurrence and initial detection times. These strains are also phylogenetically closely related to other EBHSV strains circulating in Europe, likely confirming the slow evolutionary dynamics of this lagovirus species.

## 1. Introduction

European brown hare syndrome (EBHS) was recognized for the first time in Sweden in 1980 [1], although it may have appeared earlier in Europe [2,3,4]. Since that time, outbreaks of EBHS have been reported among free-living hares in different European countries [3,5,6,7,8,9,10,11,12,13,14,15], including Poland [16,17,18]. The disease usually has an acute course with nonspecific symptoms typical of generalized viral infections and leads to sudden death [1]. It is caused by the European brown hare syndrome virus (EBHSV) which belongs to the Lagovirus genus within the *Caliciviridae* family [14,19,20]. Besides EBHSV and rabbit hemorrhagic disease virus (RHDV), the *Lagovirus* genus also includes nonpathogenic hare caliciviruses (HaCV) [21], which may have an impact on the recombination events and differentiation process of EBHSV [22,23,24]. The non-enveloped capsid of lagoviruses contains a single-stranded RNA of positive polarity, approximately 7.5 kb in length. Unlike other animal caliciviruses, the EBHSV and RHDV genomes contain two open reading frames (ORFs), the first encoding the nonstructural proteins as well as the major capsid virus protein (VP60), and the second encoding the gene of the small structural protein (VP10) [20,25,26,27].

The first genetic and phylogenetic analyses of the EBHS virus strains focused on the short fragments (265–398 bp) of the VP60 gene that encode the capsid structural protein [2,28,29]. They showed the phylogenetic distinctiveness of EBHSV from RHDV and indicated high strain similarity within each calicivirus species. Despite the small number of EBHSV sequences available, phylogenetic variability of EBHSV strains associated with their geographical origin and time of detection has been observed [29]. Analysis of complete VP60 gene sequences also disclosed temporal- and geographic-related differences and revealed the existence of two main genetic groups of EBHSV: group A, encompassing Swedish EBHSV strains detected before 1989, and group B, with virus strains detected after 1989 in the rest of Europe [30]. Contrary to these results, the analyses of partial VP60 gene fragments of approximately 200 bp, have shown at least three independent directions of the virus’ spread from Scandinavia across Europe [1,3,5,8,16,19,25,28]. This finding has confirmed the slow evolutionary dynamics of EBHSV, evidenced by low genetic variability of the strains and formation of new genetic subgroups of limited geographical range [8,28,31,32]. The variability of the EBHSV strains could be related to the evolution of RHDV, in which there have been observed recombination events between different virus types including nonpathogenic hare caliciviruses [23]. No similar type of recombination encompassing the capsid protein gene has been observed among EBHSV strains so far [30]. Nevertheless, it was mapped in the genome region encoding the nonstructural proteins, resulting in the emergence of EBHSV/RHDV2 recombinants [33].

Data on the epidemiology of EBHSV infections and strain virulence circulating in the European hare population are very limited. Gaining full understanding of these aspects of EBHSV is circumscribed by the difficulties in growing it in cell cultures. These difficulties significantly hamper research related to the assessment of the virus’ variability and recognition of the role and functions of particular genes in adaptation of the virus to new hosts.

The purposes of the study were to determine the genome nucleotide sequences of the EBHSV strains from Poland, and to identify conserved or variable single-nucleotide polymorphisms (SNPs). Viral phylogenetic relationships to previously detected EBHSV strains in Europe were also investigated. In order to determine the genetic affiliation of the Polish strains to currently identified EBHSV subgroups, a nucleotide sequence analysis of the VP60 gene was conducted. The amino acid sequences of this gene were also analyzed to identify the presence of mutations specific to the virus subgroups.

## 2. Materials and Methods

### 2.1. EBHSV Strains and Lagovirus Sequences

Five EBHSV strains (NP1192, L98, K501, G104, and K204) detected in hares from 1992 to 2004 were used for the genetic analysis. These virus strains now belong to the strain collection of the National Reference Laboratory for Rabbit Myxomatosis at the National Veterinary Research Institute in Puławy, Poland. Infected animals were found dead in different geographical locations across Poland and liver and spleen samples were taken from the carcasses. The presence of EBHSV in the animals was confirmed by testing the tissue samples using an RHDV–EBHSV CR Mab ELISA kit (IZSLER, Brescia, Italy).

For the phylogenetic analysis, 10 full EBHSV genome sequences, 46 VP60 complete EBHSV sequences, and 10 VP10 complete EBHSV sequences, available in GenBank, were used. These comprised virus strains of particular EBHSV genogroups detected in Sweden (KC832838–39, KJ679513–14, KJ679522, KJ679534–35, KJ679547–48, KJ679553, KJ679558–59, and KJ679562–63), France (AJ971301, AJ971304–06, AJ971311, AJ971315, AM408588, AM887765, AM933648–50, FN689419–21, HF571039–40, KY801206, LT168848, and Z69620), Italy (X98002, KF591083, KJ923230, KU961677–78, and MF356366) and Germany (U09199, LR899140, LR899152, LR899171, LR899182, LR899185, and LR899188). Additionally, six sequences of nonpathogenic HaCV and rabbit calicivirus (RCV) lagoviruses from France (MH204883), Italy (KR230102) and Australia (EU871528 and MK138383–85), as well as RHDV (genotype GI.1b, M67473), and two EBHSV/RHDV2 (LR899142 and LR899187) recombinant strains were also included.

### 2.2. Amplification of the EBHSV Genome

To determine the genome sequences of Polish virus strains, amplification of the overlapping genome fragments was conducted using primers specific to the EBHSV genome (Supplementary Table S1). Additionally, several sets of primers were designed based on the reference EBHSV-GD89 (Z69620) sequence to cover the missing regions of the virus genome using the primer-BLAST tool (https://www.ncbi.nlm.nih.gov, accessed on 22 September 2018). Primers were synthesized by Genomed S.A. (Warsaw, Poland). EBHSV RNA was extracted from animal tissues using an RNeasy Mini Kit (Qiagen, Hilden, Germany), according to the manufacturer’s instructions. Subsequently, for amplification of individual EBHSV genome fragments, the OneStep RT-PCR Kit, (Qiagen, Hilden, Germany) was employed. The reactions were conducted in 50 µL of PCR mixture containing 2 µL of enzyme mix, 1 × buffer, 400 µM of dNTPs, 0.6 µM of the appropriate forward and reverse primer pair, 10 U of RNAsin (Invitrogen, Waltham, MA, USA), 5 µL of RNA template and redistilled water to the required reaction volume. The following amplification protocol was used: reverse transcription at 50 °C for 30 min and initial denaturation at 94 °C for 30 s followed by 35 cycles consisting of denaturation at 94 °C for 30 s, primer annealing (from 54 °C to 58 °C depending on the primer set) (Supplementary Table S1) for 45 s, extension at 72 °C for 1 min, and a final elongation step at the same temperature but for 10 min. PCRs yielded products varying in length from 265 to 858 bp.

### 2.3. Sequencing and Phylogenetic Analyzis

PCR amplicons were visualized in 1.5% agarose gel, purified and directly sequenced in both directions using the ABI Prism BigDye Terminator v3.1 Cycle Sequencing Kit on an ABI 3730XL DNA sequencer (Life Technologies, Carlsbad, CA, USA) at the Genomed S.A. sequencing service. For comparative analysis of nucleotide sequences of EBHSV strains, BLASTn software was used (accessed on 22 January 2021) [34,35]. The sequences were aligned using Clustal W [36]. The phylogenetic trees were constructed based on 15 full genome, 51 VP60 and 15 VP10 complete gene sequences of EBHSV using a Maximum Likelihood method with the Tamura–Nei model with uniform rates, and Nearest-Neighbor-Interchange (NNI) method for topology searching in MEGA version 7.0.26 [37]. This tool has also been employed in recent studies on lagovirus phylogeny [38,39]. A branch support was estimated using 1000 bootstrap replicates. The phylogenetic relationship among the sequences analyzed was considered reliable when the bootstrap value was ≥70%. The obtained genome sequences of Polish virus strains were deposited in GenBank under accession numbers MK440613–MK440617.

## 3. Results

### 3.1. Sequence Analyzis of EBHSV Strains from Poland

The amplicons’ sequences were merged to the nearly complete genome sequences of the EBHSV L98 (7424 bp), NP1192 (7422 bp), G104 (7414 bp), K204 (7409 bp) and K501 (7330 bp) lacking only the initial twenty-nucleotide fragment upstream of the 5ʹ untranslated region. Only the K501 sequence did not contain the 3ʹUTR fragment. The highest sequence similarities in the entire genome (97.6%), the VP60 gene (97.8%), and in the nonstructural protein (NSP) gene (97.6%) were observed between the K501 and L98 strains. However, the lowest genetic resemblances corresponding to 93%, 93.4%, and 92.8% nucleotide identity in similar genome fragments were between the K204 and NP1192 strains. In the ORF2 region encoding the structural VP10 protein, 98.8% sequence similarity between EBHSV K501 and NP1192 was found despite the nine-year difference between their first detections. Although detected at similar times, K204 and G104 revealed the lowest percentage of identical nucleotides in their VP10 genome fragments at 93.6%. The similarity of the genomic nucleotide sequences of the analyzed virus strains and their fragments encoding structural and nonstructural proteins are presented in Table 1. At 98%, the highest nucleotide sequence similarity of the whole virus genome was observed between NP1192 and the GD89 (Z696290) reference EBHSV strain. When each genome fragment was analyzed separately, the sequence genetic resemblances were 99.4% (VP60), 99.4% (RNA-dependent RNA polymerase), 97.0% (NSP), and 99.1% (VP10). The other Polish virus strains were 93–94% similar to EBHSV GD89 in the whole genome. The comparison of the NP1192 ORF1–ORF2 sequence with the Swedish O4022-10 reference virus strain gave 94% sequence similarity. It ranged from 91 to 92.5% for other Polish strains, although the individual NSP, VP60, and VP10 virus segments showed a relatively higher (91–94%) sequence identity.

### 3.2. Analysis of the Lagovirus Phylogenetic Relationships Based on ORF1–ORF2 Genome Sequences

**Figure 1:**
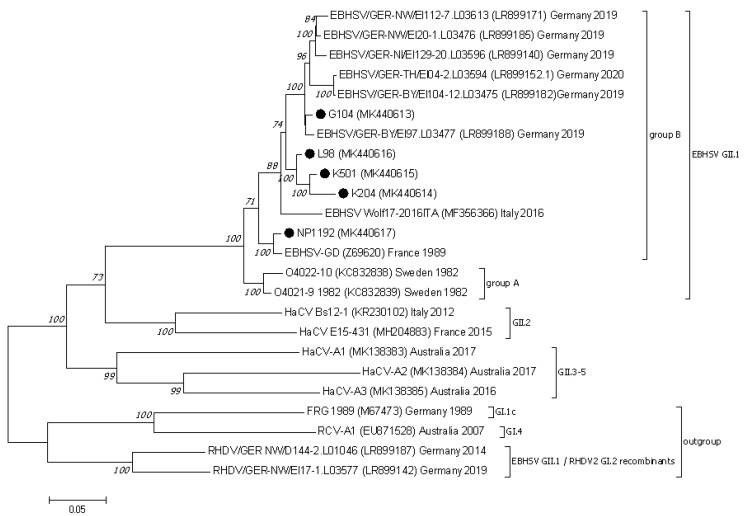
The phylogenetic Maximum Likelihood tree constructed using the ORF1–ORF2 (21–7350 bp) nucleotide sequences of European EBHSV strains and other hare and rabbit caliciviruses. Bootstrap values (1000 replicates) greater than 70% are shown at the corresponding tree nodes. The tree is drawn to scale with branch lengths in the same units as those of the evolutionary distances used to infer the phylogenetic tree. Evolutionary analyses were conducted using MEGA7 [37]. The rabbit hemorrhagic disease virus—RHDV (GI.1c), EBHSV GII.1/RHDV2 GI.2 recombinants, and rabbit calicivirus RCV–A1 (GI.4) were used as an outgroup to root the tree. EBHSV strains from Poland are marked by black dots.

The phylogenetic analysis of genome sequences of EBHSV strains encompassing the ORF1 and ORF2 region (nucleotide positions 21–7350 according to the reference EBHSV-GD (Z69620) strain) assigned them to one common cluster including strains from group A and group B (Figure 1). In the group of EBHSV strains from Poland, NP1192 clusters together with the French reference EBHSV-GD. The L98, K501, K204 and G104 EBHSV strains form one genetic cluster (bootstrap value 100%) jointly with the Italian Wolf17–2016 EBHSV sequence. Within this virus group, two subclusters are formed, one containing the three Polish strains L98, K501, and K204 and the other the G104 strain and virus sequences detected in 2019–2020 in Germany. The results of phylogenetic analysis of the whole virus genome (ORF1–ORF2) and its segments encoding the VP60 and VP10 structural proteins indicates the distinctiveness of G104 from the group of the other EBHSV strains from Poland. Nevertheless, some relatedness was observed to younger European virus strains originating from France and Germany. In the VP60 region, the nucleotide sequence differences between G104 and the other virus strains from the G1.3 and G1.1 subgroups including EBHSVs from group A did not exceed 9.7%, while they were 5% for strains clustered to the G2 group. The highest nucleotide sequence similarity of 99.4% was found between G104 and the 0836 (HF571040) French strain detected in 2008. In the case of the EBHSV strains from France, Sweden, Italy, and Germany clustered together in the G3 group, the nucleotide sequence differences were in the range of 1.3–3.5% (Supplementary Table S2).

### 3.3. Nucletide and Amino Acid Sequence Analysis of the Structural Genes of EBHSV Strains from Poland and Elsewhere in Europe

**Figure 2:**
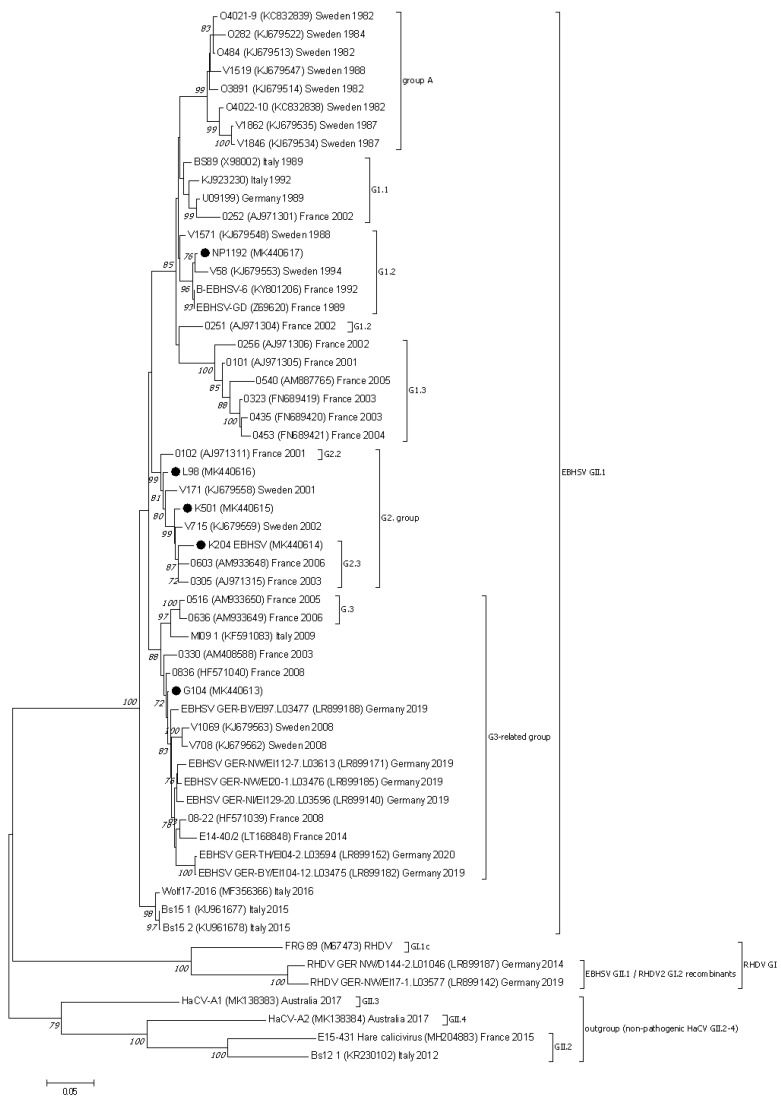
The phylogenetic Maximum Likelihood tree constructed using nucleotide sequences of the VP60 protein gene of European EBHSV strains and other hare and rabbit caliciviruses. Bootstrap values (1000 replicates) greater than 70% are shown at the corresponding tree nodes. The tree is drawn to scale with branch lengths in the same units as those of the evolutionary distances used to infer the phylogenetic tree. Evolutionary analyses were conducted using MEGA7 [37]. The nonpathogenic hare caliciviruses—HaCV (GII.2–4) were used as an outgroup to root the tree. EBHSV strains from Poland are marked by black dots.

**Figure 3:**
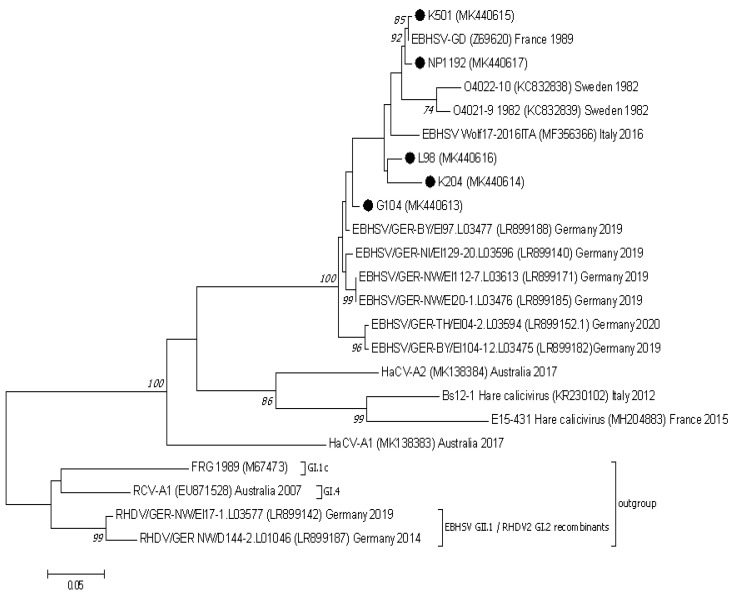
The phylogenetic Maximum Likelihood tree constructed using nucleotide sequences of the VP10 protein gene (ORF2) of European EBHSV strains and other hare and rabbit caliciviruses. Bootstrap values (1000 replicates) greater than 70% are shown at the corresponding tree nodes. The tree is drawn to scale with branch lengths in the same units as those of the evolutionary distances used to infer the phylogenetic tree. Evolutionary analyses were conducted using MEGA7 [37]. The rabbit hemorrhagic disease virus—RHDV (GI.1c), EBHSV GII.1/RHDV2 GI.2 recombinants, and rabbit calicivirus RCV-A1 (GI.4) were used as an outgroup to root the tree. EBHSV strains from Poland are marked by black dots.

The phylogenetic analysis conducted based on the VP60 and VP10 sequences showed that the EBHSV strains from Poland belonged to the G1, G2, or G3 virus genetic groups (Figure 2 and Figure 3). NP1192 and the virus strains from France and Sweden detected in a similar time span are clustered together in the G1.2 subgroup of the G1 cluster. The L98, K501 and K204 Polish strains are related to other French EBHSV strains first detected between 2000 and 2006, which are classified to the G2.2 and G2.3 subgroups. In the case of the G104 strain, it belongs to a large group of G3 EBHSV strains detected in Europe at different times and geographical locations.

The differences in the amino acid sequences between the Polish virus strains range from 1.1 to 2.6% in the entire two-open-reading-frame genome. For the genome fragment encoding the VP60 protein, the variability of the amino acid sequence is 0.9–2.7%; however, for the VP10 fragment it is higher than those observed for the entire genome, at 0.9–5.5%. In comparison to the VP60 region of the reference EBHSV-GD strain, this region of the Polish virus strains’ sequences identified 22 variable amino acids sites, i.e., 58 (A/V), 66 (V/A), 231 (D/E), 270 (S/C), 291 (I/F), 302 (S/T), 327 (I/V), 343 (T/S), 383 (S/N), 410 (T/A/S), 415 (I/L), 417 (L/M), 427 (V/T), 461 (A/S), 476 (A/S), 522 (V/I), 524 (M/L), 536 (A/T), 542 (D/E), 544 (T/A), 565 (L/F), and 566 (A/T) (Table 2). Substitutions at positions 427, 522, and 524 for NP1192 are consistent with the changes observed with EBHSV sequences from the G1 group, whereas the TIL pattern of amino acids of the remaining Polish strains corresponds to the G2 virus group. In the sequences of the Polish L98, K501, G104, and K204 strains, identical amino acid substitutions occur at eight positions, i.e., 58, 66, 302, 327, 383, 427, 522, and 524. The T410A amino acid change was present in the sequences of the L98, K501, and K204 strains, and in the T/S form also in the G104 sequence. The substitutions at positions D542E and A566T are common to the L98, G104, and K204 strains. Two other substitutions, T343S and A476S, appeared in the K501 and K204 sequences. The remaining amino acid changes occur in sequences of Polish virus strains. Analysis of the VP60 amino acid profile of the NP1192 strain at sites 302 (S), 383 (S), 406 (A), 407 (Q), 427 (V), and 469 (V) affiliate this strain to the G1.2 subgroup with the French EBHSV GD89 and B-EBHSV-6 strains. Furthermore, the analysis of codons 383, 410, and 476 located in the C-terminal part of the VP60 gene of EBHSV strains from Poland revealed concordance of the amino acid profile of the L98 strain with that of the Swedish V171 and French 0102 strains from the G2.2 subgroup. The amino acid substitutions at positions 383, 410, and 476 found in K501 and K204 are similar to those of other strains belonging to the G2.3 subgroup. A VP60 amino acid profile is observed for G104, which differs at positions 410, 417, and 476 from other Polish strains, but is consistent with the profile of the 0330 French strain from 2003 and other European EBHSV strains from Sweden, France, and Italy in the 2005–2014 period, as well as from strains detected in recent years in Germany belonging to the G3 group.

In comparison to the Z69620 VP10 reference amino acid sequence, EBHSV strains from Poland and elsewhere in Europe were characterized by changes in six amino acids. Substitutions were identified at four amino acid positions (3, 64, 94, and 113). The E3D substitution was found in the three strains L98, G104, and K204, the N64S change appeared in the pair L98 and G104, and in the N64G form also in K204. The N94S change occurred in the L98, G104, and K204 strain sequences, whereas the N113D change was present in the K501 and G104 strains. Amino acid changes in positions G60S and L85F were present in the Polish G104 strain; however, they also appeared in EBHSV strains detected in 2019 and 2020 in Germany. Analysis of the genome of nonpathogenic GII.2, GII.3, and GII.4 hare caliciviruses proved that the same change at position 60 was also present in the HaCV Bs12-1 sequence and in the G/N form also in the HaCV-A1 sequence. The L85F substitution occurred in all sequences of analyzed nonpathogenic hare caliciviruses.

## 4. Discussion

The population of hares has decreased sixfold over the last 30 years in Poland. Among the factors responsible for the significant reduction in animal numbers are heavy predation, poaching, and the occurrence of infectious diseases, with EBHSV infections being considered the main cause of death [40]. Additionally, over the whole continent of Europe, a constant decline in the populations of hares and other related species has been observed due to EBHSV outbreaks [1]. A new threat is the RHDV 2 rabbit lagovirus, which crossed the species barrier causing infections in hares with a similar course to RHD [41,42,43,44,45,46].

Although in this study a phylogenetic analysis of EBHSV strains utilizing the complete sequences of the virus genome was carried out, only the VP60-based analyses revealed deep phylogenetic relationships among EBHSV strains. In this context, EBHSV strains from Poland were clustered to the G1, G2, and G3 lineages within the larger virus group B, together with other virus strains from Europe detected in similar time spans [32]. In contrast to virus group A, which encompasses the oldest strains from the Scandinavian Peninsula which disappeared in the late 1980s, group B covers strains detected in Europe more recently [30]. These findings are consistent with previous partial VP60 sequence analyses of Polish EBHSV strains [18,47,48]. Subsequent deeper analysis of the VP60 sequence of the NP1192 strain (G1 group) confirmed its evolutionary relationships with the phylogenetically older French EBHSV-GD89 and B-EBHSV-6 strains as well as with the Swedish V58 strain belonging to the G1.2 subgroup. However, the NP1192 strain did not reveal mutations in codons 406, 407, or 469, which are characteristic of EBHSV strains in the closely related G1.3 subgroup. The four EBHSV strains L98, K501, K204, and G104 belong to the G2 and G3 groups together with French and other European EBHSVs detected since 1999. The phylogeny of these Polish virus strains was also confirmed by analysis of the amino acid sequences of the VP60 gene at the mutational hot spots (aa 427 V/T, 522 V/I, and 524 M/L) specific to G2 viruses [32]. The fifth strain, G104, is related to a much younger virus lineage consisting of Swedish and French strains discovered from 2008 to 2014, as well as to the newer German strains from 2019 to 2020. The close genetic relationships between EBHSV strains from Poland and Germany may have resulted from the geographical proximity of their detection sites and could indicate the common routes of virus spread in a given geographical area.

The profile of amino acid sequences deduced from the nucleotide sequences of the EBHSV strains from Poland in the mutational hot spot positions of VP60 reflects the division of EBHSV strains into the genetic groups previously identified among French strains [32]. The V427, V522, and M524 amino acids characteristic of the G1 group are present in the NP1192 strain. The remaining Polish strains, L98, K501, K204, and G104, contain T, I, and L amino acids in their sequences, which are typical of French, Swedish, and Italian EBHSV strains forming the G2 group as well as strains of the G3 group [30]. Despite the Polish strains described in this study belonging to different genetic virus groups, they seem to represent the same serotype, which can still be detected using currently available ELISA kits (data not shown). Although in other studies distinct antigenic profiles of EBHSV strains have been observed, which could mirror changes in VP60 physiochemical properties, the phenotypic effect was still uncertain [30]. Analysis of the ORF2 genome sequences of EBHSV strains from Poland and elsewhere in Europe from 2016 to 2020, and of nonpathogenic HaCV GII.2–4 caliciviruses, indicates the presence of mutations at amino acid positions 60 and 85 in the VP10 structural protein gene. Among the EBHSV strains from Poland, the amino acid substitutions G60S and L85F occur only in the G104 sequence from 2004. They are also characteristic of much younger EBHSV strains from Germany from 2019 to 2020, although they did not appear in the sequence of the Wolf-17 strain from Italy from 2016. These mutations reflect changes observed in the structural part of the virus genome and can be considered as indirect evidence for the evolutionary processes related to the emergence and spread of a new genetic lineage of the virus strains in the hare population in Poland. The observed variability of the amino acid sequence and results of the phylogenetic analysis of the ORF2 region, supported by the phylogeny of VP60 and the entire genome, confirm G104’s relationship to EBHSV strains circulating in Europe between 2008 and 2020. Moreover, these results support the assumption that the ORF2 region can be employed in tracking the evolution of EBHSV strains to a greater extent than previously thought.

The analysis of the shared phylogenetic resemblance of the Polish virus strains indicates their low genetic variability and likely slow evolutionary dynamics compared with other lagovirus species such as RHDV. Low genetic diversity among the European EBHSV strains has previously been observed [28,29,31,32]. The virus seems to be more conservative; however, the evolutionary process still occurs and has resulted in the disappearance of the oldest group A strains and the emergence of several virus lineages within the new genetic group B [30]. Evidence for the slow evolutionary dynamics of the virus could be the presence of only a relatively small number of new virus variants within the existing genetic subgroups. A possible slow evolution mechanism may be also supported by the close phylogenetic relationships observed between Polish strains from 2001 to 2004 and a much younger Italian EBHSV strain detected in 2016. The close genetic relationship of EBHSV strains is mainly determined by their geographical origin rather than the time span in which their first isolation was achieved [32]. Another factor which may have an impact on evolution dynamics and the emergence of new EBHSV variants are infections of hares with nonpathogenic HaCV and RHDV2, which are phylogenetically related to EBHSV. Nevertheless, the observed lower genetic variability among EBHSV strains in comparison to RHDV could also be associated with the smallness of the hare population limiting the frequency of events of interspecies transmission of the virus and could be associated with the possible natural resistance of those species to EBHSV infection [1,10,15,31,32,40]. It must be noted that based on ORF1-ORF2 sequence analysis of Polish RHDV variants and EBHSV strains, there were no recombination events observed between these two virus species (unpublished data). Likewise, the analyses of the VP60 and VP10 EBHSV sequences, as well as available full genome sequences of lagoviruses, did not reveal any evidence confirming this recombination phenomenon in the group of Polish EBHSV strains. Furthermore, they formed a separate phylogenetic branch in relation to pathogenic RHDV (GI.1) and RHDV2 (GI.2), as well as to nonpathogenic RCV-A1 (GI.4), which were clustered as outgroup sequences.

In addition, there is no vaccine available against EBHSV infection, so mutation is not driven by vaccine-related selective pressure on the population of wild virus strains [1,32]. This situation significantly diminishes the risk of the appearance of virus variants able to evade the host’s immune system. Nevertheless, the detection of new genetic subtypes of nonpathogenic hare and rabbit caliciviruses in Europe and Australia emphasizes the role of asymptomatic infections in fostering the genetic variability of lagoviruses [38,39], and it can be assumed that these infections could enhance the further differentiation of EBHSV strains. A recombination mechanism analogous to that previously observed for nonpathogenic RCV and RHDV2 could also differentiate EBHSV [23,38,42]. The main limitation of each study aiming to analyze the genetic variability of EBHSV is the small number of full genome sequences of the virus available. It also hinders the assessment of their phylogenetic relationships and the investigation of lagovirus evolutionary paths. Nevertheless, a certain degree of genetic diversity among Polish strains was established, allowing for their classification to the G1, G2 or G3 genetic group.

## 5. Conclusions

EBHSV strains from Poland confirmed their close phylogenetic relationship to other EBHSV strains circulating in Europe. The results of this retrospective study also provide evidence that they show high genetic resemblance despite their different occurrence locations and detection time spans.

## Figures and Tables

**Table 1:** The nucleotide sequence analysis of the genomes of Polish, reference and other European EBHSV strains.

EBHSV Strain/Acc. Number	NP1192				L98				K501				G104				K204			
	ORF1-ORF2	NSP	VP60	VP10	ORF1-ORF2	NSP	VP60	VP10	ORF1-ORF2	NSP	VP60	VP10	ORF1-ORF2	NSP	VP60	VP10	ORF1-ORF2	NSP	VP60	VP10
	* 21–7330	21–5282	5282–7013	7006–7330	21–7330	21–5282	5282–7013	7006–7330	21–7330	21–5282	5282–7013	7006–7330	21–7330	21–5282	5282–7013	7006–7330	21–7330	21–5282	5282–7013	7006–7330
NP1192/MK440617																				
L98/MK440616	94.7	94.6	94.8	96.5																
K501/MK440615	94.1	93.7	94.3	98.8	97.6	97.6	97.8	95.9												
G104/MK440613	94.5	94.4	94.7	94.2	96.5	96.6	96.4	95.4	95.5	95.5	95.7	94.8								
K204/MK440614	93.0	92.8	93.4	94.8	96.0	95.8	96.6	95.9	97.0	96.9	97.5	94.8	94.5	94.4	95.2	93.6				
EBHSV-GD **/Z69620	97.7	97.1	99.4	99.1	94.3	94.0	94.9	96.2	93.7	93.1	94.2	99.7	94.0	93.7	94.9	94.5	92.7	92.3	93.5	95.1
O4022-10 **/KC832838	93.7	93.7	93.6	94.2	92.5	92.6	92.1	92.8	91.7	91.7	91.3	94.2	91.9	92.2	91.4	91.3	91.0	91.0	90.7	92.2
WOLF17/MF356366	93.5	93.2	94.0	96.4	95.0	94.8	95.5	95.7	94.3	93.9	94.8	96.7	94.8	94.7	95.3	94.2	93.4	93.0	94.2	95.8
L03596/2019/LR899140	93.5	93.2	94.3	94.2	95.2	95.3	95.4	94.2	94.3	94.2	94.5	94.2	97.5	97.3	98.3	97.7	93.4	93.1	94.6	92.5
L03594/2020/LR899152	92.7	92.5	93.6	92.2	94.6	94.6	95.1	92.2	93.8	93.6	94.6	92.3	96.4	96.3	97.1	95.7	92.9	92.6	94.2	90.7
L03613/2019/LR899171	93.6	93.4	94.0	93.6	95.4	95.4	95.6	93.9	94.5	94.3	95.2	93.6	97.4	97.3	98.0	97.4	93.4	93.1	94.5	92.8
L03475/2019/LR899182	92.8	92.6	93.7	92.2	94.6	94.6	95.3	92.2	93.8	93.6	94.8	92.3	96.5	96.3	97.2	95.7	92.9	92.5	94.3	90.7
L03476/2019/LR899185	93.8	93.6	94.3	93.6	95.6	95.8	95.8	93.9	94.6	94.5	95.4	93.6	97.8	97.5	98.5	97.4	93.6	93.3	94.9	92.8
L03477/2019/LR899188	94.2	94.1	94.5	94.5	96.0	96.4	95.6	95.1	95.0	95.1	94.9	94.5	98.5	98.4	98.7	98.6	94.0	94.0	94.5	93.3

* nucleotide positions according to EBHSV-GD (Z69620); ** reference EBHSV strains. ORF1-ORF2—open reading frame 1 and 2; NSP—nonstructural protein genes; VP60—capsid structural protein; VP10—minor capsid structural protein.

**Table 2:** Amino acid substitutions in the VP60 region of EBHSV strains from Poland (compared to the reference EBHSV-GD 89 strain and representatives of the EBHSV genetic groups).

EBHSV Strain	Accession Number	Origin	Genetic Group/Subgroup	Position of Amino Acids in Particular Regions of VP60 Gene																					
				b					c	d	e						f								
				58	66	231	270	291	302	327	343	383	410	415	417	427	461	476	522	524	536	542	544	565	566
EBHSV-GD	Z69620	France	B/G1.2	A	V	D	S	I	S	I	T	S	T	I	L	V	A	A	V	M	A	D	T	L	A
NP1192	MK440617	Poland 1992	B/G1.2	A	V	D	S	I	S	I	T	S	T	I	L	V	A	A	V	M	A	D	T	L	A
L98	MK440616	Poland 1998	B/G2.2	V	A	D	S	F	T	V	T	N	A	I	L	T	A	A	I	L	A	E	A	L	T
K501	MK440615	Poland 2001	B/G2.3	V	A	D	S	I	T	V	S	N	A	I	L	T	A	S	I	L	A	D	T	L	A
K204	MK440614	Poland 2004	B G2.3	V	A	D	S	I	T	V	S	N	A	L	L	T	S	S	I	L	T	E	T	F	T
G104	MK440613	Poland 2004	B/G3	V	A	E	C	I	T	V	T	N	S	I	M	T	A	A	I	L	A	E	T	L	T
BS89	X98002	Italy 1989	B/G1.1	A	V	D	S	I	T	I	T	S	S	I	L	V	A	V	V	M	A	D	T	L	A
V58	KJ679553	Sweden 1994	B/G1.2	A	V	D	S	I	T	I	T	N	T	I	M	V	A	V	V	M	A	D	T	L	A
A0256	AJ971306	France 2002	B/G1.3	A	V	D	S	I	T	V	S	N	T	L	L	V	A	S	V	M	T	E	T	F	T
V171	KJ679558	Sweden 2001	B/G2.2	A	A	D	S	I	T	V	T	N	A	I	L	T	A	A	I	L	T	E	T	L	T
V715	KJ679559	Sweden 2002	B/G2.3	V	A	D	S	I	T	V	S	N	A	I	L	T	A	S	I	L	A	E	T	L	A
O516	AM933650	France 2005	B/G3	A	V	E	C	I	T	I	T	N	S	I	M	T	A	A	I	L	A	E	T	L	A
MI09	KF591083	Italy 2009	B/G3	A	V	E	C	I	T	I	T	N	S	L	L	T	A	A	I	L	A	E	T	L	T
BS15-1	KU961677	Italy 2015	B/-undefined	A	A	D	S	I	T	V	S	N	S	I	M	V	A	A	I	L	A	E	T	L	T
WOLF	MF356366	Italy 2016	B/-undefined	A	A	D	S	I	T	V	S	N	S	I	M	V	A	A	I	L	A	E	T	L	T
0330	AM408588	France 2003	B/G3	V	A	E	C	I	T	I	T	N	S	I	M	T	A	A	I	L	A	E	T	L	T
E14-40/2	LT168848	France 2014	B/G3	A	A	E	C	I	T	V	T	N	S	I	M	T	A	A	I	L	A	E	T	L	T
08-36	HF571040	Sweden	B/G3	V	A	E	C	I	T	V	T	N	S	I	M	T	A	A	I	L	A	E	T	L	T
L03596/2019	LR899140	Germany	B/G3	A	A	E	C	I	T	V	S	N	S	I	M	T	A	A	I	L	A	E	T	L	T
L03594/2020	LR899152	Germany	B/G3	A	A	E	C	I	T	V	T	N	S	I	M	T	A	A	I	L	A	E	T	L	T
L03613/2019	LR899171	Germany	B/G3	A	A	E	C	I	T	V	S	N	S	I	M	T	A	A	I	L	T	E	T	L	T
L03475/2019	LR899182	Germany	B/G3	A	A	E	C	I	T	V	T	N	S	I	M	T	A	A	I	L	A	E	T	L	T
L03476/2019	LR899185	Germany	B/G3	A	A	E	C	I	T	V	S	N	S	I	M	T	A	A	I	L	A	E	T	L	T
L03477/2019	LR899188	Germany	B/G3	A	A	E	C	I	T	V	T	N	S	I	M	T	A	A	I	L	A	E	T	L	T
O4021-9	KC832839	Sweden 1982	A	A	M	D	S	I	N	I	T	N	S	I	L	V	A	D	V	M	A	D	T	L	A

## Data Availability

Sequences determined in this study were submitted to GenBank database under the accession numbers MK440613–MK440617.

## Supplementary Materials

The following are available online at https://www.mdpi.com/article/10.3390/v13101999/s1, Table S1: Primers used for amplification and sequencing of the overlapping EBHSV genome fragments. Nucleotide positions (5′–3′) refer to the genome sequence of EBHSV-GD89 strain (GenBank accession no. Z69620). Table S2: Evolutionary divergence between VP60 sequences of EBHSV, nonpathogenic HaCV, RHDV, and RHDV2 including EBHSV/RHDV2 recombinant strains. Evolutionary analyses were conducted in MEGA7 [37].
