# Supplementary material for: Phylogenetic Analysis of European Brown Hare Syndrome Virus Strains from Poland (1992–2004)
Source: Viruses. 2021 Oct 5;13(10):1999. doi: 10.3390/v13101999 (PMC8539919; doi:10.3390/v13101999)
Supplement: Supplementary file 1 [file viruses-13-01999-s001.zip › Supplementary Table S1.pdf]

Supplementary Table S1.

Primer's name (Forward / Reverse)	Genome position	Nucleotide sequence (5'-3')	Amplicon length (bp)	Annealing temperature (°C)	Reference
0001F	0001-0020	GTGAAATTATGGCGGTTGCG	695	52	[49]
27R	0695-0676	ATCCTGCAGGGCCAATTTCA			This study
17F	0375-0397	AAGGTCATCCCTTGCTTGCACTC	747	55	This study
1085R	1104-1085	GGACTGAGTCCTCAAACTTG			[49]
0908F	0908-0929	CCTGCTGGATTGGACAAATGA	600	55	[49]
29R	1507-1487	TTCCCAAGTACGAGCGTGTGG			This study
18F	1416-1441	AGGTGCACACTCCCGTCGTACAACCA	581	57	This study
1977R	1997-1977	GAAGGTGAGGTGAGACATGTC			[49]
18F	1416-1441	AGGTGCACACTCCCGTCGTACAACCA	794	57	This study
30R	2210-2187	CTGTTTCCACATTAAAAGTGCTTG			This study
1761F	1761-1782	CCTCTCAACTGTGACAAGGTTG	625	56	[49]
31R	2386-2367	ACGCAACCAAGTGCAAAGTG			This study
19F	1908-1927	AACAAGGCCGTAGAAGGGTG	858	56	This study
2745R	2766-2745	GGTCACCAGAGTATCTCACAAG			[49]
20F	2289-2308	GCGAGGAAGTCAGATCCCAG	808	56	This study
32R	3097-3076	TCAAGGAATTCCTCCGCTGTCA			This study
54F	2341-2360	TGAAGTACATTTGGAGGATG	810	55	This study
55R	3169-3188	TGAAGTACATTTGGAGGATG			This study
2615F	2615-2634	CAATCCGGTGTGTGCATATG	675	54	[49]
33R	3290-3271	ACCACCGTCGTCAAGAGTTC			This study
EBHS-1F	2633-2652	TGACACGCTAATTCGCACTC	517	54	This study
EBHS-1R	3149-3130	AAACTTGATGGCGTCTGCAT			This study
21F	3128-3147	TGATGCAGACGCCATCAAGT	728	56	This study
3835R	3856-3835	CCTGGAACCTTTGCATACCTTG			[49]
3767F	3767-3786	CATTGACTACCGTGGACTTG	856	57	[49]
4604R	4623-4604	GAACGGTCATTGGAAGTGAC			[49]
4503F	4503-4522	GATTCCACGATGTCACCATG	836	57	[49]
5320R	5339-5320	AGGAACAGATGCTGTGGTAG			[49]
22F	5274-5293	GTGAATGTTATGGAGGGCAA	408	56	[50]
34R	5682-5658	TGATTGCACACACAAGCCTTCCGCC			[50]
VP60_0001F	5283-5305	ATGGAGGGTAAGCCWCGGGCTGA	532	56	[49]
35R	5814-5795	CTGTTGGGTGGTACATTTCA			This study
23F	5671-5690	TGTGTGCAATCATACCGCCT	495	54	This study
VP60_0885R	6166-6147	CTTGGATGRTCAATGTCGTC			[30]
VP60_0813F	6095-6115	CAGRCACTGGAAYATGAATGG	592	55	[30]
HEB	6687-6666	CATCACCAGTCCTCCGCACCAC			[2]
HEF	6423-6445	CCGTCCAGCATTCGTCCTGTCAC	265	56	[2]
HEB	6687-6666	CATCACCAGTCCTCCGCACCAC			[2]
HEF	6423-6445	CCGTCCAGCATTCGTCCTGTCAC	587	54	[2]
VP60_1728R	7010-6987	GACATAGGAATATCCAGTGGTGGC			[49]
6911F	6911-6929	GACAGACCTCATTGACGTG	531	58	[49]
dTNN	polyA	GACTGACTGCCATGGCCGGCGCTAGC TTTTTTTTTTTTTTTTTTTTTTTTT			[51]
46F	6874-6893	ATGCAGGGACTGGTGCTTAT	569	58	This study
dTNN	poly A	GACTGACTGCCATGGCCGGCGCTAGC TTTTTTTTTTTTTTTTTTTTTTTTT			[51]
